# Expression of DISC1-Interactome Members Correlates with Cognitive Phenotypes Related to Schizophrenia

**DOI:** 10.1371/journal.pone.0099892

**Published:** 2014-06-18

**Authors:** Antonio Rampino, Rosie May Walker, Helen Scott Torrance, Susan Maguire Anderson, Leonardo Fazio, Annabella Di Giorgio, Paolo Taurisano, Barbara Gelao, Raffaella Romano, Rita Masellis, Gianluca Ursini, Grazia Caforio, Giuseppe Blasi, J. Kirsty Millar, David John Porteous, Pippa Ann Thomson, Alessandro Bertolino, Kathryn Louise Evans

**Affiliations:** 1 Medical Genetics Section, Centre for Genomic and Experimental Medicine, Medical Research Council Institute of Genetics and Molecular Medicine, The University of Edinburgh, Edinburgh, United Kingdom; 2 Department of Neuroscience and Sense Organs, University of Bari, Bari, Italy; 3 Istituti di Ricovero e Cura a Carattere Scientifico Casa Sollievo della Sofferenza, Section of Liaison Psychiatry, San Giovanni Rotondo (FG), Italy; 4 Centre for Cognitive Ageing and Cognitive Epidemiology, Medical Genetics Section, Centre for Genomic and Experimental Medicine, Medical Research Council Institute of Genetics and Molecular Medicine, University of Edinburgh, Edinburgh, United Kingdom; 5 Imaging, Pharma Research and Early Development, Neuroscience Discovery and Translational Area, Hoffman-La Roche, Basel, Switzerland; Louisiana State University Health Sciences Center, United States of America

## Abstract

Cognitive dysfunction is central to the schizophrenia phenotype. Genetic and functional studies have implicated *Disrupted-in-Schizophrenia 1* (*DISC1*), a leading candidate gene for schizophrenia and related psychiatric conditions, in cognitive function. Altered expression of *DISC1* and DISC1-interactors has been identified in schizophrenia. Dysregulated expression of DISC1-interactome genes might, therefore, contribute to schizophrenia susceptibility via disruption of molecular systems required for normal cognitive function. Here, the blood RNA expression levels of *DISC1* and DISC1-interacting proteins were measured in 63 control subjects. Cognitive function was assessed using neuropsychiatric tests and functional magnetic resonance imaging was used to assess the activity of prefrontal cortical regions during the N-back working memory task, which is abnormal in schizophrenia. Pairwise correlations between gene expression levels and the relationship between gene expression levels and cognitive function and N-back-elicited brain activity were assessed. Finally, the expression levels of *DISC1*, *AKAP9*, *FEZ1*, *NDEL1* and *PCM1* were compared between 63 controls and 69 schizophrenic subjects. We found that DISC1-interactome genes showed correlated expression in the blood of healthy individuals. The expression levels of several interactome members were correlated with cognitive performance and N-back-elicited activity in the prefrontal cortex. In addition, *DISC1* and *NDEL1* showed decreased expression in schizophrenic subjects compared to healthy controls. Our findings highlight the importance of the coordinated expression of DISC1-interactome genes for normal cognitive function and suggest that dysregulated *DISC1* and *NDEL1* expression might, in part, contribute to susceptibility for schizophrenia via disruption of prefrontal cortex-dependent cognitive functions.

## Introduction

Abnormal cognitive function is recognized as a core component of the schizophrenia phenotype [1], [2]. Cognitive deficits are often apparent before the onset of positive symptoms, are heritable [3], and are seen in unaffected relatives of patients [4]. Moreover, while deficits in working memory are a good predictor of functional outcome [5], cognitive deficits are often refractory to current drug treatments [6].

A network of brain regions, including the prefrontal cortex (PFC), contributes to cognition [7]. Several of these regions demonstrate abnormal activity in schizophrenia [8]. Schizophrenic patients are impaired in the performance of PFC-dependent tasks such as the N-back task, a test of working memory [9]. This task elicits a heritable pattern of activity in the PFC [3], meaning it can be used to assess whether “schizophrenia-risk” polymorphisms exert their pathogenic effect by altering PFC function.

Variation in *Disrupted in Schizophrenia 1* (*DISC1*) is a risk-factor for schizophrenia [10], [11] and is implicated in cognitive function in both healthy individuals and schizophrenic patients [12].

The DISC1 protein interacts with several proteins to link multiple cellular functions and molecular pathways, including neuronal migration (LIS1, NDE1, NDEL1, Dixdc1), axonal bundling and elongation (FEZ1), neural progenitor proliferation (GSK3β), cell cycle regulation (PCM1), neuronal signalling (Girdin, GSK3β, PDE4), and signal transduction (Kal7, TNIK, AKAP9) [10], [13]. Polymorphisms in several genes encoding DISC1-interactors have been associated with schizophrenia [10], [14], and altered hippocampal expression of *NDEL1*, *FEZ1*, and *LIS1* has been detected in post-mortem brains of schizophrenic patients [15]. Thus, alterations in brain function that confer risk for psychiatric illness might arise from dysfunction at several loci in the DISC1-interactome. In support of this assertion, Hennah and Porteous [16] have shown genetic variation in *DISC1* and DISC1-interacting proteins to modulate RNA expression of genes involved in schizophrenia-relevant processes, including cytoskeletal functions, synaptogenesis, neurodevelopment and sensory perception. This finding is particularly pertinent in light of the evidence for the involvement of perturbed gene expression in schizophrenia [17].

In this study, we test the hypotheses that: (i) genes encoding DISC1-interactome members show coordinated expression; (ii) expression levels of these genes are associated with cognitive function; (iii) frontal cortical activity elicited by the N-back task is related to the expression of genes whose expression level is associated with cognitive function; and (iv) the expression of genes whose expression correlates with cognitive function is abnormal in schizophrenic patients. We demonstrate associations between: (i) the expression levels of several DISC1-interactome members; (ii) DISC1-interactome members’ expression levels and cognitive performance; and (iii) genes whose expression is related to cognitive performance and frontal cortical activity. Moreover, we find the expression of *DISC1* and *NDEL*, whose expression levels correlate with cognitive performance, to be reduced in the blood of schizophrenic patients.

## Materials and Methods

### Ethics Statement

Procedures were carried out in accordance with The Code of Ethics of the World Medical Association (Declaration of Helsinki) and approval was given by the local ethics committee (“Comitato Etico Indipendente Locale-Azienda Ospedaliera Ospedale Policlinico Consorziale di Bari”), who examined the study protocol and the suitability of the environment and investigators involved. Participants gave written informed consent. The capacity of participants to consent was assessed by a psychiatrist. If participants were not deemed capable of giving informed consent, a legal representative gave consent on their behalf. In accordance with Articles 414 and 415 of the Civil Code, the Legal Representative was a member of the patient’s family, a clinician or a lawyer nominated by the Local Court in order to represent the patient’s civil rights.

### Subjects

We examined 63 control subjects (age: mean = 26.2, standard deviation (SD) = 5.4; 36 females) and 69 subjects with schizophrenia (age: mean = 37.3, SD = 10.8, 16 females). Control subjects were screened using the Non-Patient Structured Clinical Interview for DSM-IV to ensure they were unaffected by any psychiatric condition. In addition, control subjects were screened using the Family Interview for Genetic Studies [18] to ensure absence of psychotic disorders in their first-degree relatives. Diagnosis of schizophrenia was made using the Structured Clinical Interview for the DSM-IV Axis 1 disorders, which was administered by psychiatrists. Both patients and control subjects were excluded if they had: a significant history of drug or alcohol abuse; active drug abuse in the previous year; experienced a head trauma with a loss of consciousness; or if they suffered from any other significant medical condition.

### Gene Expression Levels

Blood for RNA extraction was collected using the Tempus Blood Collection System (Applied Biosystems). RNA was extracted from 3 ml whole blood using Tempus Blood RNA Isolation Kits and subsequently reverse transcribed using Transcriptor First Strand cDNA Synthesis Kits (Roche Applied Science). mRNA expression was measured by quantitative reverse-transcription polymerase chain reaction (qRT-PCR), using TaqMan probe-based assays (Applied Biosystems; see Table S1 in File S1 for probe IDs). Reactions were performed using an ABI Prism 7900 Sequence Detection System (Applied Biosystems), at the Wellcome Trust Clinical Research Facility, Edinburgh. Assays spanned an exon-exon boundary and amplified the maximum possible number of isoforms. Expression levels were calculated using the relative standard curve method and gene-of-interest expression levels (*DISC1*, *AKAP9*, *FEZ1*, *GSK3β*, *LIS1*, *NDE1*, *NDEL1*, *PCM1*, *PDE4B*, *PDE4D*) were normalised to the geometric mean of two reference genes (*RPLPO* and *HNRNPD*). These reference genes were selected on the basis of geNorm analysis [19], which showed these genes to be stably expressed in a representative sub-set of the blood samples assessed in this study (N = 15 schizophrenic patients and 15 controls). Each sample was measured in technical triplicate. Between-plate differences were accounted for by normalisation to a calibrator. Outliers were identified and excluded following Burns et al. [20].

### Cognitive Assessment

All subjects were assessed with a battery of cognitive tests comprising the: (i) N-Back paradigm (2-back condition); (ii) Continuous Performance Test, assessing sustained attention; (iii) Wechsler Memory scale, giving a memory quotient; (iv) Wisconsin Card Sorting Test, evaluating executive functions; (v) Trail Making A and B, assessing cognitive flexibility; and (vi) Controlled Oral Word Association Test (Benton, 1967), measuring Semantic Fluency and Phonological Fluency. For further details, see File S1 Materials and Methods.

### Gene-by-gene Expression Interactions

Pearson pairwise correlation coefficients were calculated for each pair of gene expression levels using SPSSv17.0 software (www.spss.com). To control for the effects of multiple testing, permutation analysis (10^5^ permutations) was performed using Resampling (http://core.ecu.edu/psyc/wuenschk/StatHelp/Resampling.htm, East Carolina University) which adopts a classical “randomization without replacement” algorithm. The same software was used to derive 95% bootstrap confidence intervals for correlation coefficients. Bootstrapping was conducted by running a total of 10^5^ re-samples drawn pairwise from our original sample. Corrected *p*≤0.05 indicated statistical significance.

### Association between Gene Expression and Cognitive Performance

#### Principal component analysis

Principal component analysis (PCA) on a correlation matrix followed by varimax rotation, retaining factors with an eigenvalue ≥1 was used to group cognitive functions. In addition to providing an insight into the architecture of cognitive function, this approach reduces the number of dependent variables, thus reducing the type 1 error rate [21], [22].

#### Correlation of gene expression with cognition

Multiple regression analysis with backward stepwise removal was conducted for the expression levels of ten genes against the scores obtained for each of the four principal components (PCs). The variable with the smallest partial correlation with the dependent variable was considered for removal first, and removed if the significance level of its F-value was *p*>0.05. For all PCs, a higher score indicated better performance. For each analysis, the model with the highest R^2^ and the lowest *p*-value was taken to best explain the variance within the relevant PC. All gene expression levels and their pairwise interactions were then regressed within each model against the relevant PC. The threshold for statistical significance was *p*≤0.05. Analyses were conducted using SPSSv.17.0.

### fMRI Paradigm

The fMRI experimental paradigm consisted of the N-back task, as described previously [23]. The stimuli consisted of numbers (1–4) shown in random sequence and displayed at the points of a diamond-shaped box. A non-memory-guided control condition (0-back) presented the same stimuli but simply required subjects to identify the current stimulus. In the working memory condition, the task required recollection of a stimulus seen two stimuli previously (2-back). Performance data were recorded as the percentage of correct responses and reaction time (msecs).

Echo planar imaging blood oxygenation level dependent fMRI data were acquired as described previously [23] (TE = 30 m secs, TR = 2 secs, 20 contiguous slices, voxel size = 3.75×3.75×5) on a GE 3T scanner with a standard head coil. A simple block design was used in which each block consisted of 8 alternating 0-back and 2-back conditions (each 30 secs), obtained in 4 min and 8 secs, 120 whole-brain fMRI volumes. The first four scans of the time series were discarded to allow for signal equilibration effects.

### fMRI Data Analysis

Images were pre-processed using Statistical Parametric Mapping 8 software (SPM8; http://www.fil.ion.ucl.ac.uk/spm). Images were realigned to the first volume in the time series to correct for head motion (<2 voxels translation, <1° rotation), re-sampled to a 2 mm isotropic voxel size, spatially normalized into a standard stereotactic space (Montreal Neurological Institute template) using a 12 parameter affine model and smoothed to minimize noise and residual differences in gyral anatomy with a Gaussian filter, set at 6 mm full-width at half-maximum. Voxel-wise signal intensities were ratio normalized to the whole-brain global mean. For each experimental condition, a box-car model convolved with the haemodynamic response function at each voxel was modelled. Pre-determined condition effects at each voxel were calculated using a t-statistic, producing a statistical image for the contrast of 2-back versus 0-back. Individual contrast images were then used in second-level random effects models to determine task-specific regional responses at the group level for the entire sample.

To assess the association between gene expression and fMRI activation, contrast images of all subjects were used as dependent variables in a general linear model using SPM8. A priori evidence for involvement of the frontal cortex in the N-back task [3], [9] meant this region was of particular interest. Regions of interest (ROIs) were specified by dividing the frontal cortex into its constituent Brodmann Areas (BAs), as defined by the WFU_PickAtlas [24]. Within each ROI, a family wise error (FWE) small volume correction with a *p*≤0.05 was applied to control for type I errors.

### Comparison of Gene Expression Levels in Patients with Schizophrenia and Healthy Controls

Case-control comparisons were carried out for *AKAP9*, *DISC1*, *FEZ1*, *NDEL1* and *PCM1* expression levels. To assess the influence of potentially confounding variables, the effects of age and gender on expression were assessed for each gene using one-way analysis of variance (ANOVA) and linear regression analysis, respectively. Comparisons of gene expression levels between schizophrenic patients and controls were made using either ANOVA or analysis of covariance (ANCOVA) with the independent variable of diagnostic status and the dependent variable of expression level. Covariates were included where appropriate.

## Results

### Gene-by-gene Expression Interactions

In the control subjects, we assessed correlations between RNA expression levels of *DISC1* and nine DISC1-interactors by calculating Pearson correlation coefficients for each pair-wise combination. Following correction for multiple testing by permutation and bootstrap analyses, *DISC1* expression showed significant correlation with *AKAP9*, *GSK3β*, and *NDEL1*. Several other significant pair-wise correlations were detected (Table 1; Figure S1 in File S1).

**Table 1:** Gene co-expression within the DISC1-interactome.

Co-expressedgene pair	Pearsoncorrelationcoefficient (*r*)	Uncorrectedp*-*value(2-tailed)	Corrected*p*-value^a^	95% bootstrapConfidenceinterval
DISC1 × AKAP9	+0.27	0.046	0.048	0.012; 0.53
DISC1 × GSK3β	−0.35	0.0082	0.012	−0.55; −0.34
DISC1 × NDEL1	+0.29	0.036	0.036	0.038; 0.51
AKAP9 × FEZ1	+0.42	0.0033	0.0052	0.14; 0.42
AKAP9 × PCM1	+0.39	0.0032	0.0052	0.036; 0.39
FEZ1 × NDE1	−0.45	0.0012	0.0012	−0.68; −0.18
NDE1 × GSK3β	+0.57	0.000033	0.000055	0.37; 0.72
NDE1 × NDEL1	+0.32	0.033	0.033	0.003; 0.56
GSK3β × PDE4B	+0.32	0.023	0.041	0.11; 0.32

a Corrected by permutation analysis (10^5^ permutations).

### Principal Component Analysis of Cognitive Test Data

**Figure 1:**
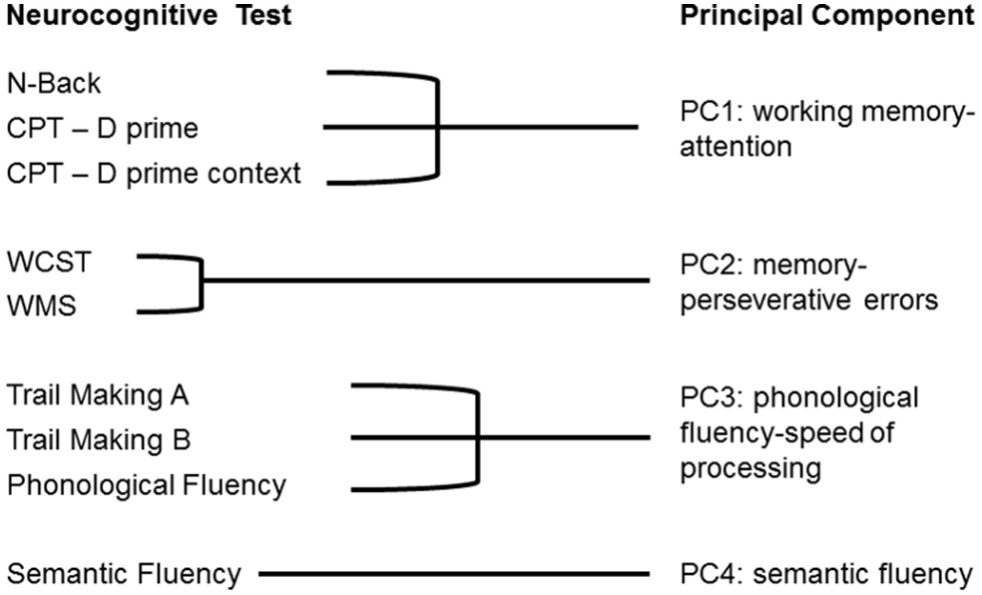
Graphical depiction of the principal components derived from performance on the neurocognitive tests. Four principal components were defined by principal components analysis. Each principal component was named according to the specific cognitive functions assessed by the tests that loaded onto it.

As we had carried out multiple related cognitive tests we defined a set of linearly independent cognitive variables using principal component analysis (PCA). PCA revealed four principal components (PCs) that together explained 66.5% of the variance in cognitive performance (*p* = 0.003; Figure 1; Table S2 in File S1).

### Correlation between Gene Expression and Cognitive Performance

To explore the correlation between gene expression and cognition we carried out backward removal multiple regression analysis of gene expression levels against the four PCs. For each PC, variation in the expression of either a single gene or a combination of genes was found to explain a significant proportion of the variance (Table 2). We next examined the effect of gene-by-gene interactions within PCs 2, 3 and 4 (PC1 was best explained by a single-gene model). Backward removal multiple regression analysis revealed that inclusion of interaction terms improved the fit and significance of each model (Table 3; Figure S2 in File S1). The combination of genes and gene×gene interactions that best explained variation in each PC will henceforth be referred to as the “expression model”.

**Table 2:** Relationship between the expression levels of DISC1-interactome genes and cognitive performance.

PC^a^	Cognitivefunctions assessed	Model^b^	βcoefficient	*p*-value	Wholemodel*p*-value	Wholemodel R^2^
1	Working Memory –Attention	NDEL1	+0.34	0.02	0.023	0.12
2	Memory -Perseverative Errors	AKAP9FEZ1PCM1	+0.48−0.47−0.38	0.0020.0020.01	0.0014	0.35
3	Phonological Fluency - Speed of Processing	DISC1NDEL1	+0.28−0.38	0.0720.015	0.029	0.16
4	Semantic Fluency	DISC1AKAP9	−0.26+0.41	0.0670.002	0.0052	0.23

a PC = principal component.

b The model explaining the most variation within each PC was determined by backward removal multiple regression analysis including main effects only.

**Table 3:** Relationship between the expression levels of DISC1-interactome genes and their pairwise interactions and cognitive performance.

PC^a^	Model^b^	βcoefficient	*p*-value	Whole model*p*-value	Wholemodel R^2^
2	AKAP9FEZ1PCM1FEZ1 × PCM1	+0.41−0.45−0.34−0.65	0.00280.00210.0150.000019	0.00038	0.38
3	NDEL1DISC1NDEL1 × DISC1	−0.39+0.33−0.65	0.0040.0140.0058	0.0052	0.21
4	AKAP9DISC1AKAP9 × DISC1	+0.47−0.26−0.28	0.000460.0380.0003	0.0013	0.23

a PC = principal component.

b The model explaining the most variation within each PC was determined by backward removal multiple regression analysis including main effects and gene-by-gene interaction terms.

### Correlation between Gene Expression and Frontal Cortical Function

We assessed the relationship between the four expression models and N-back-elicited frontal cortical activity, as measured by fMRI, in the control subjects. Several findings at the fMRI level were consistent with findings at the behavioural level (Table 4; Table S3 in File S1).The PC 2, 3, and 4 expression models showed significant relationships with the activity of frontal regions (Figures S3 and S4 in File S1). The PC1 expression model showed a nominally significant relationship with activity in BA 9 (uncorrected *p*<0.0001); however, this relationship did not withstand multiple testing correction (corrected *p* = 0.093).

**Table 4:** Relationship of gene expression levels to activity in the prefrontal cortex elicited by the N-back task.

PC^a^	Regression term(gene/gene×geneinteraction)	Brodmann areashowing significantlycorrelated activity	Direction ofcorrelation	Corrected*p*-value^b^	Whole model*p*-value
1	NDEL1	9	POSITIVE	0.093	0.093
2	AKAP9PCM1FEZ1FEZ1 × PCM1	9454545	NEGATIVENEGATIVENEGATIVEPOSITIVE	0.0350.0290.0400.026	0.01
3	NDEL1DISC1NDEL1 × DISC1	666	NEGATIVENEGATIVEPOSITIVE	0.0250.0230.022	0.00083
4	AKAP9DISC1AKAP9 × DISC1	999	POSITIVEPOSITIVENEGATIVE	0.0190.0070.009	0.00078

a PC = principal component.

b Corrected by permutation analysis (10^5^ permutations).

### Comparison of the Expression Levels of DISC1-Interactome Members in Schizophrenic Patients and Healthy Controls

The expression levels of five members of the DISC1-interactome whose expression was correlated with cognitive performance in control subjects (*AKAP9*, *DISC1*, *FEZ1*, *NDEL1*, and *PCM1*) were measured in the peripheral blood of schizophrenic patients. As age significantly affected *DISC1* expression (*p* = 0.012), it was included as a covariate in the DISC1 analysis. Comparing schizophrenic patients to controls revealed significant differences in DISC1 (*p* = 0.0015, ANCOVA) and NDEL1 (*p* = 0.028, ANOVA) expression. Both genes showed reduced RNA expression in schizophrenic patients (*DISC1*: mean = 1.33, SD = 0.810; *NDEL1*: mean = 1.88, SD = 0.294) relative to controls (*DISC1*: mean = 2.10, SD = 1.20; *NDEL1*: mean = 2.15, SD = 0.936).

## Discussion

Multiple lines of evidence support a role for variation in *DISC1* and DISC1-interactors in conferring risk for psychiatric illness [10], [11], potentially, in part, via an effect on cognitive function [12]. DISC1’s role as a molecular scaffold [10] suggests that the coordinated expression of DISC1-interactome members is essential for normal brain development and function, and that alteration may result in cognitive deficits and susceptibility to psychiatric illness.

Here, in healthy individuals, we demonstrated significant correlations between the expression levels of several members of the DISC1-interactome. Moreover, RNA expression of five of these genes (*AKAP9*, *DISC1*, *FEZ1*, *NDEL1*, and *PCM1*) correlated with cognitive performance. Cognitive function, as assessed by several measures, was reduced to four PCs. The expression level of at least one DISC1-interactome gene was significantly correlated with each PC. Interestingly, where multiple genes best explained performance, the majority of these genes showed significant between-gene correlations in their expression levels. It is noteworthy that genes showing correlated expression often conferred opposite effects on cognitive function. Furthermore, the inclusion of interaction terms improved the fit and the significance of the models. These findings are suggestive of a complex scenario whereby members of gene pairs showing correlated expression contribute to cognitive function via both independent and overlapping mechanisms. Our findings suggest that it is unlikely that genes showing correlated expression contribute to the same cognitive PC via a mechanism that simply involves one gene regulating the expression of the other and, thus, conferring an effect on cognition. Nevertheless, it would be of interest in the future to perform experiments *in vitro* to establish whether manipulating the expression level of one member of a correlated gene pair alters the expression of the other member of the gene pair. Our results indicate the inter-dependency of DISC1-interactome members and highlight the possibility for variation in their expression levels to confer risk for schizophrenia via a deleterious effect on cognition.

We report that N-back task-elicited activity in BAs 9, 45, and 6 is correlated with the PC1, PC2, PC3, and PC4 expression models. Our observation of N-back task-elicited activity in these regions is consistent with previous studies [25]. The expression models for PCs 1, 2, and 4 correlated with activity in BAs 9 and 45, regions that have been implicated in working memory function [25], [26]. BA6 has traditionally been viewed as motor cortex; however, the observation that this area is active in non-motor mental operation tasks that require the updating of spatial and verbal representations in memory suggests it may subserve both cognitive and motor functions [27], [28]. PC3, the cognitive model for which expression correlated with BA6 activity, related to phonological fluency and speed of processing. Interestingly, activity in BA6 has previously been found to correlate with response rate on a word fluency task [29].

Within each expression model, the effect of each gene on N-back task-elicited activity depended on the expression of the other DISC1-interactome genes in the model and the specific brain region considered. Furthermore, significant correlations were observed between several gene-by-gene interaction terms and frontal cortical activity. For genes contributing to PCs 2, 3 and 4, the relationship between gene expression and N-back task-elicited activity was in the opposite direction for the gene-by-gene interaction terms and the related main effects. This pattern of results is compatible with a scenario whereby genes contributing to a given PC act via both mutually dependent and independent pathways, which confer opposite effects on the activity of a given brain region in response to changes in gene expression. These findings support a functional interaction between DISC1-interactome members, adding to our understanding of their known cellular interactions, and highlight the need to consider genetic variables in the context of their appropriate biological networks. Moreover, we are the first to report a correlation between the expression of a gene in the blood and frontal cortical activity.

Of the five genes whose expression correlated with cognitive function in healthy controls, *DISC1* and *NDEL1*, showed reduced expression in schizophrenic patients. This finding is in keeping with the positive correlation between *DISC1* and *NDEL1* expression observed in control subjects. In light of this result, one possibility is that the reduced expression of both genes in schizophrenic patients reflects the inter-dependency of the expression levels of these genes. This possibility could be assessed *in vitro* by manipulating the expression level of each gene individually and measuring the expression of the other gene.

Our study is the first to compare the peripheral blood expression of *NDEL1* between patients and controls and is consistent with a previous study, which demonstrated reduced NDEL1 plasma activity in schizophrenia [30]. In contrast to our findings, a recent study found *DISC1* RNA expression to be increased in peripheral blood mononuclear cells (PBMCs) from treatment-naive patients with schizophrenia and to remain increased following six to eight weeks of antipsychotic treatment [31]. The discrepancy with our findings is, therefore, unlikely to be attributable to treatment effects, with the caveat that antipsychotic treatment may take longer than eight weeks to alter *DISC1* expression. Another potential explanation for this discrepancy is the difference in the cellular composition (whole blood vs. PMBCs) of the RNA source. Our finding of reduced *DISC1* expression is consistent with our previous work [32], where we observed ∼50% reduction in DISC1 protein expression in lymphoblastoid cell lines derived from carriers of at (1;11) translocation that disrupts *DISC1* and is a strong genetic risk factor for psychiatric illness [33]. Further studies are required to characterise the relationship between blood expression of *DISC1* and schizophrenia.

Assaying expression in peripheral blood rather than post-mortem brains avoids the confounds of agonal conditions and post-mortem delay, which can affect RNA integrity and thus gene expression [34], [35]. Cognisant of these caveats, it should be noted that Lipska et al. [15] found the hippocampal mRNA expression of *NDEL1* but not *DISC1* to be reduced in schizophrenic patients. In contrast, ∼75 kDa DISC1 hippocampal protein expression was found to be increased in schizophrenic patients [15]. A recent study identified decreased 91 kDa DISC1 immunoreactivity in the dorsolateral prefrontal cortex (DLPFC) in schizophrenic patients [36], while studies of *DISC1* mRNA expression in this region have found no differences between schizophrenic patients and controls [15], [37]. There are several possible explanations for the apparent discrepancies between these studies: schizophrenia-associated changes in gene expression might be isoform, tissue- and/or brain region- specific; and/or aetiological heterogeneity might have resulted in systematic between-study sample differences. It is clear that further studies are required to distinguish between these possibilities.

While the relevance of blood expression to brain expression is a subject of debate, the use of blood RNA is a practical necessity when conducting within-subjects studies of gene expression and cognition. Additional studies in which blood and brain expression are compared within-samples would aid in the interpretation of blood-based expression findings; however, regardless of the relationship between blood and brain expression levels, our findings provide encouragement for the possibility raised by previous studies that blood-based gene expression profiling may eventually aid in diagnosis [38], [39].

In summary, this study highlights the contribution of the coordinated expression of DISC1-interactome genes to cognitive functions relevant to schizophrenia. Future studies should attempt to clarify the specificity of the relationship between the expression levels of DISC1-interactome members and sub-domains of cognitive function in independent samples of healthy controls, as well as attempting to replicate our findings of aberrant expression in schizophrenic patients. In addition it would be of interest to (i) assess the relationship between the expression of *DISC1* and *NDEL1* and cognitive function in schizophrenic patients and (ii) identify the molecular processes affected by the altered expression of these genes. Such studies have the potential to both identify new targets for therapeutic action, thus permitting the development of medications with greater efficacy in ameliorating the cognitive deficits of schizophrenia, and identify blood-based biomarkers, thus improving diagnosis.

## Supporting Information

File S1 Table S1, Details of TaqMan probes used for qRT-PCR. List of TaqMan (Applied Biosystems) probes used to explore expression levels in ten genes of the DISC1 interactome. Probes were chosen to span an intron-exon boundary and to amplify the maximum possible number of isoforms possible. Table S2, Factor loadings of cognitive tasks. Factors with eigenvalues of at least 1 were identified by principal components analysis followed by varimax rotation. For each cognitive test, the function explored is detailed together with the loading of the test on each of the four identified factors. Table S3, Assessment of the effect of gene expression levels on prefrontal cortical activity elicited by the N-back task. Correlations were assessed between gene expression and activity in prefrontal cortex during the N-back task (2-back condition). For each correlation, the Brodmann’s area (BA) involved, Talairach coordinates, k values, z-score, and familywise error (FWE) rate corrected *p*-value are indicated. Where a *p*-value did not survive multiple testing correction, this is indicated and the uncorrected *p*-value is presented. Figure S1, Patterns of co-expression amongst DISC1 pathway genes. Graphical representation of the co-expression patterns amongst DISC1 and nine DISC1-interactor genes. Lines between pairs of genes represent significant positive (continuous lines) and negative (dashed lines) Pearson correlations. Line width represents a visual proxy of correlation strength, as indicated. Figure S2, Heat map representations of the relationship between the expression of DISC1 pathway genes and cognitive performance. Depicted are heat maps representing the relationship between A. changes in PC3 (Phonological Fluency-Speed of Processing) in relation to changes in expression levels of DISC1 and NDEL1, and B. changes in PC4 (Semantic Fluency) in relation to changes in expression levels of DISC1 and AKAP9. Figure S3, Relationship between DISC1 and NDEL1 expression levels and frontal cortex function. The relationship between frontal cortical activity elicited by the N-back task and the expression levels of NDEL1, DISC1, and the interaction between NDEL1 and DISC1 expression was assessed using the general linear model. A. Heat map graphical representation of changes in fMRI BOLD signal in BA 6 during the N-back task in relation to changes in expression levels of DISC1 and NDEL1. B. Negative main effect of NDEL1 expression level on BA 6 activity during the N-back task when including NDEL1 expression level, DISC1 expression level and the DISC1-by-NDEL1 interaction term in the model. C. Negative main effect of DISC1 expression level on BA 6 activity during the N-back task when including NDEL1 expression level, DISC1 expression level and the NDEL1-by-DISC1 interaction term in the model. D. Positive effect of the interaction term, DISC1-by-NDEL1 expression level, on BA 6 activity during the N-back task when including NDEL1, DISC1, and the DISC1-by-NDEL1 interaction term in the model. Figure S4, Relationship between AKAP9 and DISC1 expression levels and prefrontal cortex function. The relationship between frontal cortical activity elicited by the N-back task and the expression levels of AKAP9, DISC1, and the interaction between AKAP9 and DISC1 expression was assessed using the general linear model. The model included AKAP9 expression level, DISC1 expression level and the AKAP9-by-DISC1 interaction. A. Heat map graphical representation of changes in fMRI BOLD signal in BA 6 during the N-back task in relation to changes in expression levels of DISC1 and AKAP9. B. Positive main effect of DISC1 expression level on BA 9 activity during the N-back task when including AKAP9 expression level, DISC1 expression level and the DISC1-by-AKAP9 interaction term in the model. C. Positive main effect of AKAP9 expression level on BA 9 activity during the N-back task when including AKAP9 expression level, DISC1 expression level and the DISC1-by-AKAP9 interaction term in the model. D. Negative effect of the AKAP9-by-DISC1 interaction on BA 9 activity during the N-back task when including AKAP9, DISC1 and the DISC1-by-AKAP9 interaction term in the model.
(DOC)
