# Supplementary material for: Expression of DISC1-Interactome Members Correlates with Cognitive Phenotypes Related to Schizophrenia
Source: PLoS One. 2014 Jun 18;9(6):e99892. doi: 10.1371/journal.pone.0099892 (PMC4062455; doi:10.1371/journal.pone.0099892)
Supplement: File S1 — Table S1, Details of TaqMan probes used for qRT-PCR. List of TaqMan (Applied Biosystems) probes used to explore expression levels in ten genes of the DISC1 interactome. Probes were chosen to span an intron-exon boundary and to amplify the maximum possible number of isoforms possible. Table S2, Factor loadings of cognitive tasks. Factors with eigenvalues of at least 1 were identified by principal components analysis followed by varimax rotation. For each cognitive test, the function explored is detailed together with the loading of the test on each of the four identified factors. Table S3, Assessment of the effect of gene expression levels on prefrontal cortical activity elicited by the N-back task. Correlations were assessed between gene expression and activity in prefrontal cortex during the N-back task (2-back condition). For each correlation, the Brodmann’s area (BA) involved, Talairach coordinates, k values, z-score, and familywise error (FWE) rate corrected p-value are indicated. Where a p-value did not survive multiple testing correction, this is indicated and the uncorrected p-value is presented. Figure S1, Patterns of co-expression amongst DISC1 pathway genes. Graphical representation of the co-expression patterns amongst DISC1 and nine DISC1-interactor genes. Lines between pairs of genes represent significant positive (continuous lines) and negative (dashed lines) Pearson correlations. Line width represents a visual proxy of correlation strength, as indicated. Figure S2, Heat map representations of the relationship between the expression of DISC1 pathway genes and cognitive performance. Depicted are heat maps representing the relationship between A. changes in PC3 (Phonological Fluency-Speed of Processing) in relation to changes in expression levels of DISC1 and NDEL1, and B. changes in PC4 (Semantic Fluency) in relation to changes in expression levels of DISC1 and AKAP9. Figure S3, Relationship between DISC1 and NDEL1 expression levels and frontal cortex f [file pone.0099892.s001.doc]

**Expression of DISC1-interactome members correlates with cognitive phenotypes related to schizophrenia**

**Materials and Methods**

**Assessment of cognitive performance**

Described below are the tasks administered to participants to assess their performance on a range of cognitive measures.

**N-back**: Working memory was assessed with the N-back test (1). ‘‘N-back’’ refers to how far back in the sequence of stimuli the subject had to recall. The stimuli consisted of numbers shown in random sequence and displayed at the points of a diamond-shaped box. There was a visually paced motor task which also served as a non-memory guided control condition (0-Back) that simply required subjects to identify the stimulus currently seen. In the working memory conditions, the task required recollection of a stimulus seen one (1-Back) or two stimuli (2-Back) previously while continuing to encode additionally incoming stimuli. Performance data were recorded as the number of correct responses (accuracy) and as reaction time.

**AX-CPT**: the AX-Continuous Performance Test measures a person’s context processing ability (2). Subjects had to press a mouse button following the presentation for 250 ms on a computer screen of a target (a letter “X”) which had been preceded by a cue (the letter “A”). Performance data were recorded as percent correct responses, reaction time, omission and commission errors. Two measures that have been found to be most sensitive to attentional processing deficits in individuals with schizophrenia are DP and DPC. DP is an index of signal detection sensitivity and it is computed using two types of false alarms: "BX" trials, in which an invalid cue (i.e., non-A) precedes the target and "AY" trials, in which a valid cue is followed by a non-target probe (i.e., non-X). DPC is computed using just “BX” trials and it provides a more specific index of sensitivity to context.

**WMS:** Wechsler Memory Scale (WMS) is used to assess short-term memory and attention functions using both auditory and visual stimuli (3). The test consists of some subtests that evaluate information, orientation, mental control, attention, logical and figural memory. WMS provides a memory quotient. More specifically, the scale includes the following subtests:

1. Information (I): six questions on general knowledge.
2. Orientation (0): five questions for orientation in time and place.
3. Mental Control (MC): the ability to repeat sequences such as the alphabet.
4. Logical Memory (LM): immediate repetition of short stories presented aurally.
5. Digits Forwards (DF), Digits Reversed (DR): the conventional digit span tests.
6. Visual Reproduction (VR): reproduction by drawing of three simple designs, each presented individually for 10 seconds.
7. Associate Learning (AL): the patient is given three trials to learn 10 pairs of words; six are obvious (up-down), and four difficult (cabbage- pen) (Wechsler, 1945).

**Wisconsin Card Sorting Test (WCST)** evaluates abstract thinking, planning, and ability to alter mental set as circumstances require (4). This is a well-established measure of general executive function. The test uses stimulus and response cards (64-card scoring version) that show various forms in various colors and numbers. Individually administered, it requires the subject to sort the cards according to different principles (i.e., by color, form, or number). As the test progresses, there are unannounced shifts in the sorting principle which require the subject to alter his or her approach. Performance data were recorded as number of correct categories and number of perseverative errors.

**Trail Making Test (TMT)** consists of two versions: TMT-version A (TMA-A) and TMT-version B (TMB-B). TMT-A is a measure of visuomotor sequencing involving connection of consecutive numbers randomly arranged on a page (circles numbered 1–25). Starting with number 1, subjects connect all circles successively, as quickly as possible, by a single continuous line, or ‘‘trail’, without lifting the pencil. TMT-B requires the connection of numbers and letters in alternating order. In particular, there are 13 randomly designated circles are numbered 1–13, and the remaining 13 are lettered A to L. Subjects draw a line connecting all 24 circles, beginning with 1, and proceeding in the order 1, A, 2, B, 3, C, etc. A practice session preceded the experimental session. For both parts A and B, the score is the total time in seconds required to complete the test. Both versions reflect attention, visual scanning, visuo-spatial memory, working memory for the target sequence, but TMT-B demands more working memory (keeping two target sequences ready instead of one) and also reflects cognitive flexibility and/or maintaining sets (5). In addition, TMT B-A differences or TMT B/A ratio scores are proposed to be indicators of executive control function (6, 7).

The **Controlled Oral Word Association Test (COWAT)** is a measure of a person's ability to make verbal associations to specified letters (i.e., A, F. and S) or semantic categories (i.e. animals, colors, fruits and places). The subject was instructed to produce as many words as possible over a one minute period for phonologic fluency or two minutes for semantic fluency. The phonologic fluency trial stipulates that no proper names or places can be used. The semantic fluency trial stipulates that no words with similar prefixes or root words can be accepted. The resulting score was the mean of all admissible words that fit within the phonologic or semantic category.

**SOM Materials and Methods References**

1. Callicott JH, Mattay VS, Bertolino A, Finn K, Coppola R, Frank JA, et al (1999): Physiological characteristics of capacity constraints in working memory as revealed by functional MRI. *Cereb* Cortex 9:20-26.

2. Braver TS, Barch DM, Cohen JD (1999): Cognition and control in schizophrenia: a computational model of dopamine and prefrontal function. *Biological psychiatry* 46:312-328.

3. Wechsler D (1945): A Standardized Memory Scale for Clinical Use. *The Journal of Psychology: Interdisciplinary and Applied* 19:87-95.

4. Berg EA (1948): A simple objective technique for measuring flexibility in thinking. *The Journal of general psychology* 39:15-22.

5. Reitan RM (1958): Validity of the Trail Making Test as an indication of organic brain damage. *Perceptual and Motor Skills* 8:271-276.

6. Arbuthnott K, Frank J (2000): Trail making test, part B as a measure of executive control: validation using a set-switching paradigm. *Journal of clinical and experimental neuropsychology* 22:518-528.

7. Perianez JA, Rios-Lago M, Rodriguez-Sanchez JM, Adrover-Roig D, Sanchez-Cubillo I, Crespo-Facorro B, et al (2007): Trail Making Test in traumatic brain injury, schizophrenia, and normal ageing: sample comparisons and normative data. *Arch Clin Neuropsychol* 22:433-447.

**SOM Tables**

**Table S1.**

| **Gene Name** | **Probe** |
| --- | --- |
| AKAP9 | Hs00183352_m1 |
| PCM1 | Hs00196390_m1 |
| NDEL1 | Hs01092620_m1 |
| FEZ1 | Hs00363763_m1 |
| DISC1 | Hs00962133_m1 |
| NDE1 | Hs00214339_m1 |
| GSK3-Beta | Hs00275656_m1 |
| PDE4B | Hs00963643_m1 |
| PDE4D | Hs00277264_m1 |
| LIS1 (PAFAHB1) | Hs00181182_m1 |

**Table S2.**

| **Task** | **Function explored** | **Factor 1** | **Factor 2** | **Factor 3** | **Factor 4** |
| --- | --- | --- | --- | --- | --- |
| Wisconsin Card Sorting Scale | Perseverative errors | -0.015 | 0.843 | 0.044 | 0.0754 |
| N-Back | Working Memory | 0.651 | -0.119 | 0.081 | -0.017 |
| Wechsler Memory Scale | Memory | 0.230 | -0.569 | 0.259 | 0.457 |
| Controlled Oral Word Association Test | Phonological Fluency | -0.001 | -0.242 | 0.773 | -0.085 |
| Controlled Oral Word Association Test | Semantic Fluency | -0.002 | 0.026 | 0.009 | 0.944 |
| Trail making A | Trail making A – Speed of processing | -0.203 | -0.228 | -0.714 | -0.0115 |
| Trail making B | Trail making B – Speed of processing | -0.257 | 0.045 | -0.689 | -0.214 |
| CPT– D prime | Attention | 0.886 | 0.058 | 0.189 | 0.089 |
| CPT – D prime context | Working Memory | 0.857 | -0.041 | 0.141 | 0.033 |

**Table S3.**

**SOM Figures**

**Figure S1.**


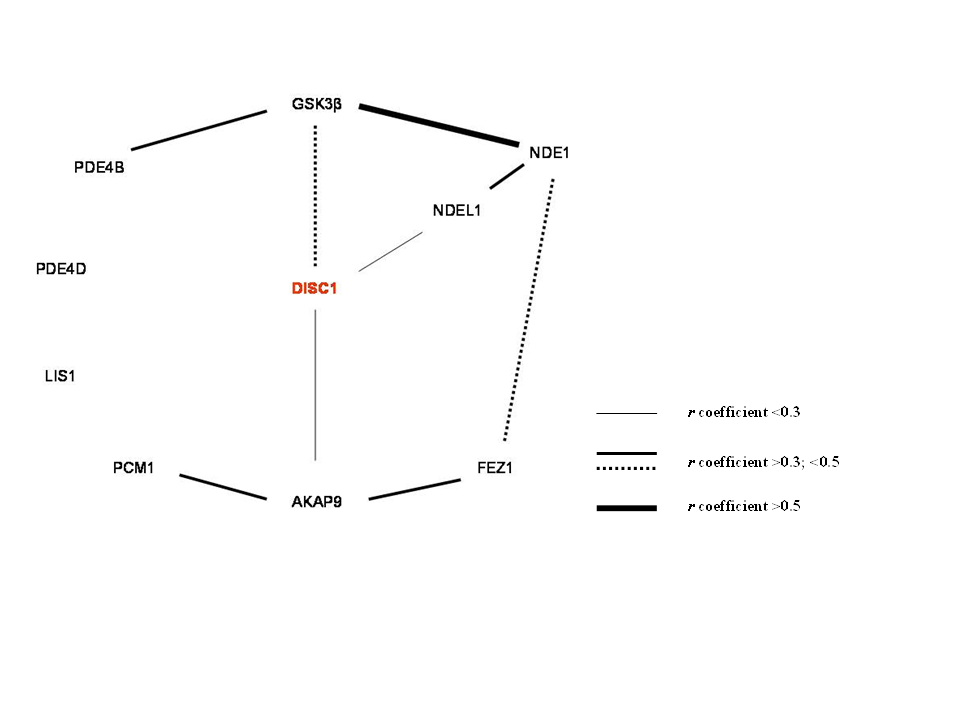


**Figure S2:**


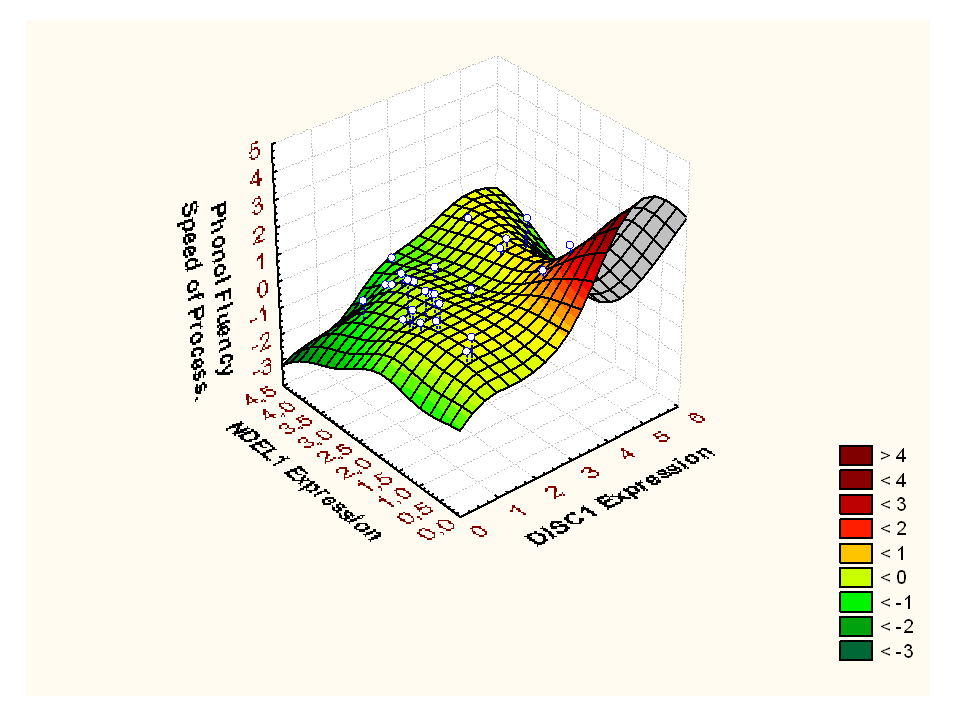


**A**


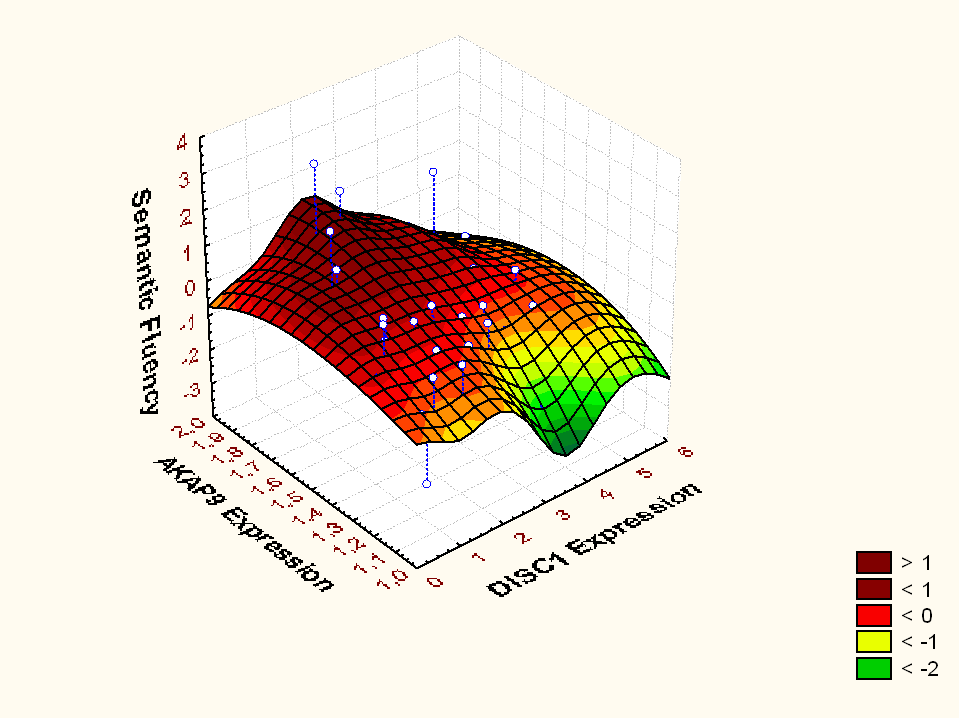


**B**

**Figure S3:**

**A**


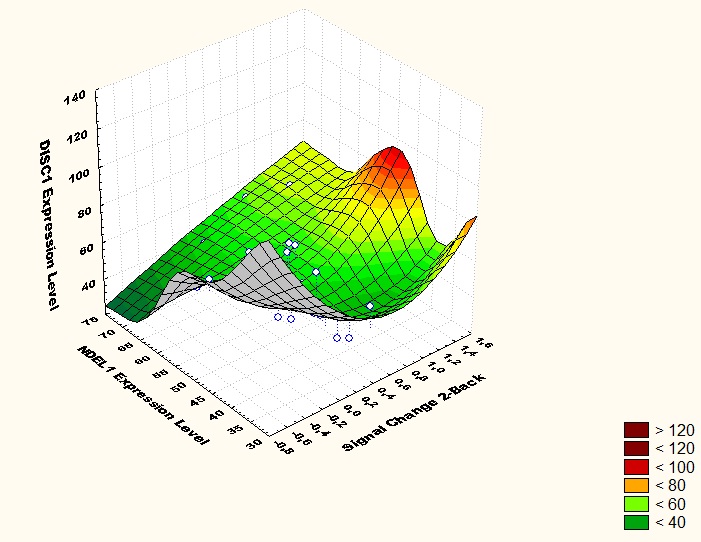


**B**

| 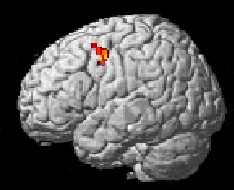 | 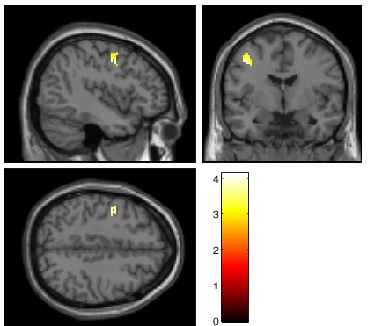 |
| --- | --- |
|  | **Left middle frontal gyrus BA 6;**  **x, y, z= -42, -4, 44; k=82; Z=3.70**  **FWE-corrected *p* = 0.025** |

**C**

| **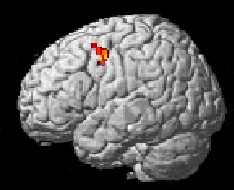** | **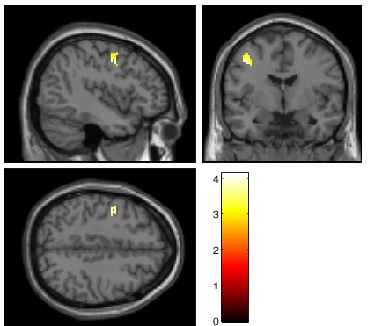** |
| --- | --- |
|  | **Left middle frontal gyrus BA 6;**  **x, y, z= -42, -4, 44; k=82; Z=3.70**  **FWE-corrected *p* = 0.023** |

**D**

| 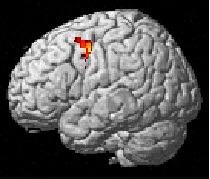 | 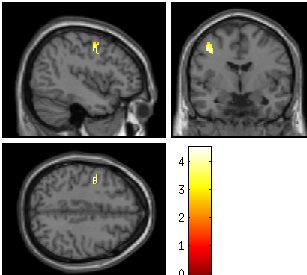 |
| --- | --- |
|  | **Left middle frontal gyrus BA 6;**  **x, y, z= -42, -4, 44; k=17; Z=3.98**  **FWE-corrected *p* = 0.009** |

**Figure S4.**

**A**


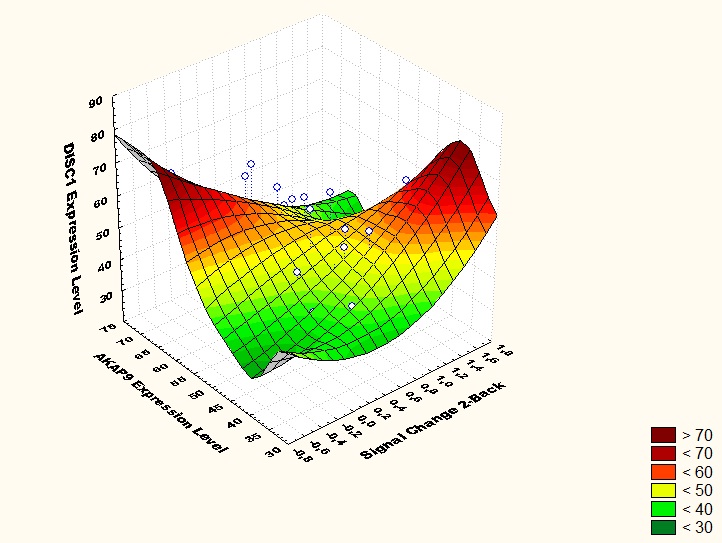


**B**

| 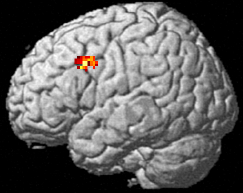 | 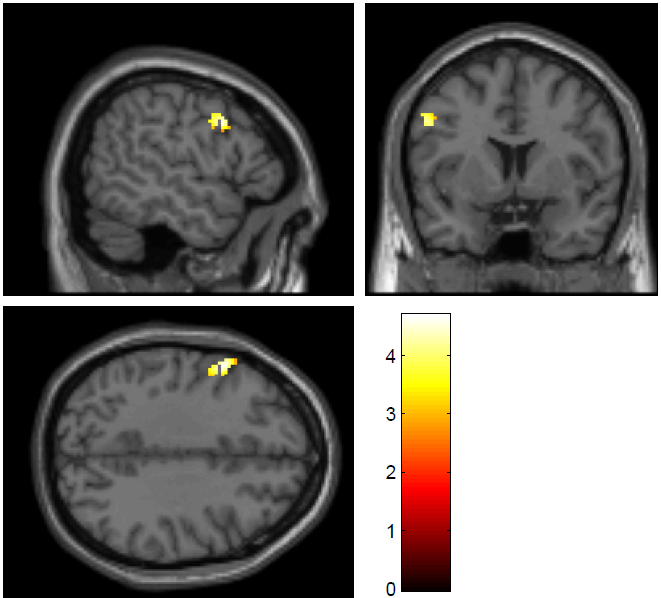 |
| --- | --- |
|  | **Left middle frontal gyrus BA 9;**  **x, y, z= -54, 10, 36; k=134; Z=4.08**  **FWE-corrected *p* = 0.007** |

**C**

| 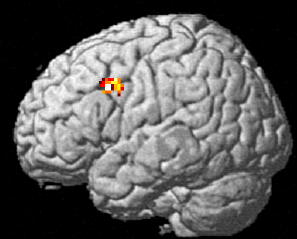 | 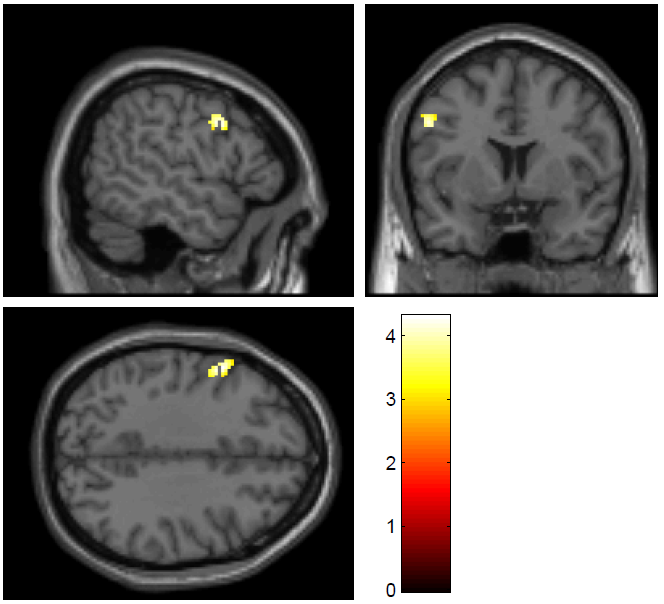 |
| --- | --- |
|  | **Left middle frontal gyrus BA 9;**  **x, y, z= -54, 10, 36; k=113; Z=3.81**  **FWE-corrected *p* = 0.019** |

**D**

| 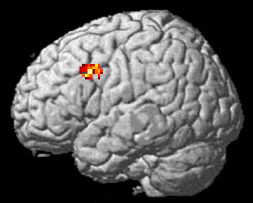 | 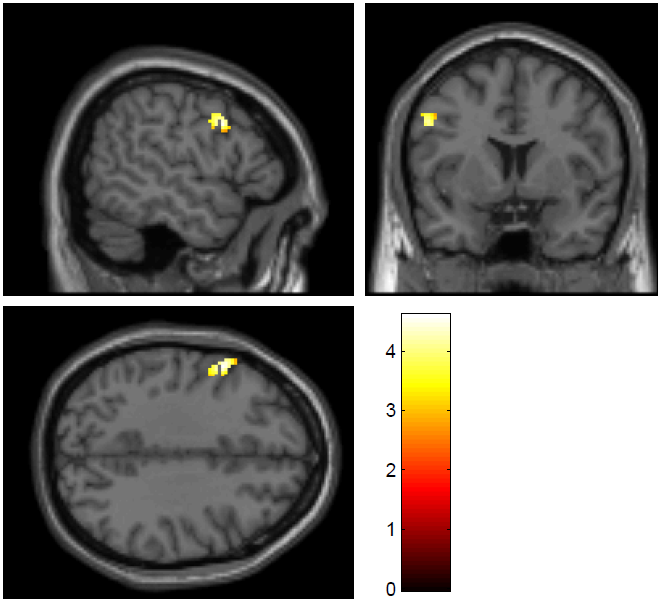 |
| --- | --- |
|  | **Left middle frontal gyrus BA 9;**  **x, y, z= -54, 10, 36; k=145; Z=4.02**  **FWE-corrected *p* = 0.009** |

**References**

1. Callicott JH, Mattay VS, Bertolino A, Finn K, Coppola R, et al (1999): Physiological characteristics of capacity constraints in working memory as revealed by functional MRI. *Cereb Cortex* 9:20-26.

2. Braver TS, Barch DM, Cohen JD (1999): Cognition and control in schizophrenia: a computational model of dopamine and prefrontal function. *Biological psychiatry* 46:312-328.

3. Wechsler D (1945): A Standardized Memory Scale for Clinical Use. *The Journal of Psychology: Interdisciplinary and Applied* 19:87-95.

4. Berg EA (1948): A simple objective technique for measuring flexibility in thinking. *The Journal of general psychology* 39:15-22.

5. Reitan RM (1958): Validity of the Trail Making Test as an indication of organic brain damage. *Perceptual and Motor Skills* 8:271-276.

6. Arbuthnott K, Frank J (2000): Trail making test, part B as a measure of executive control: validation using a set-switching paradigm. *Journal of clinical and experimental neuropsychology* 22:518-528.

7. Perianez JA, Rios-Lago M, Rodriguez-Sanchez JM, Adrover-Roig D, Sanchez-Cubillo I, et al (2007): Trail Making Test in traumatic brain injury, schizophrenia, and normal ageing: sample comparisons and normative data. *Arch Clin Neuropsychol* 22:433-447.
